# Engaging Leadership: How to Promote Work Engagement?

**DOI:** 10.3389/fpsyg.2021.754556

**Published:** 2021-10-27

**Authors:** Wilmar Schaufeli

**Affiliations:** ^1^Department of Psychology, Utrecht University, Utrecht, Netherlands; ^2^Department of Psychology, KU Leuven, Leuven, Belgium

**Keywords:** engaging leadership, work engagement, self-determination theory, employee engagement, transformational leadership

## Abstract

This paper introduces the notion of engaging leadership and reviews the empirical work done so far. Engaging leadership is defined as leadership behavior that facilitates, strengthens, connects and inspires employees in order to increase their work engagement. It can be measured with a reliable and valid self-report scale. As predicted by Self-Determination Theory, on which the concept of engaging leadership is based, basic need satisfaction mediates the relationship between engaging leadership and work engagement. This is true both for individual employees as well as the team level. In addition, job characteristics (job demands and job resources) seem to play a similar mediating role, just as personal resources. Furthermore, research shows that engaging leadership has a beneficial effect on individual and team performance which illustrates its relevance for organizations. Future research should focus, amongst others, on the opposite of engaging leadership (i.e., disengaging leadership) and interventions to foster engaging leadership. Moreover, alternative affective, cognitive and behavioral pathways should be explored that might play a role in addition to the motivational (through need fulfillment) and material (through job characteristics) pathways that have been investigated so far.

## Introduction

It is a truism that people are the most important capital for organizations, especially in today's knowledge-intensive service economies. Hence the abundant use of the notion “human capital” and the current emphasis on sustainable employability in HRM. After all, organizations should cherish their valuable and expensive human capital. Work engagement is an important indicator of sustainable employability; when employees are engaged, workability is secured (Van der Klink et al., 2016). For example, a Finnish study (Airila et al., 2012) showed that after 10 years the level of workability of engaged firefighters was significantly higher than that of their less engaged colleagues, also after controlling for lifestyle (i.e., alcohol use, smoking, exercise, sleep and BMI).

If engagement is so important to organizations—as illustrated in section Work Engagement—then the question arises how organizations can promote work engagement of their members to a higher level. This is where leadership comes into the picture. One of the principal responsibilities of leaders is to motivate their followers so that they will perform well. And because work engagement lies at the core of employee motivation, the logical question is: how can leaders promote work engagement? Answering this question is important both theoretically as well as practically, because on the one hand it provides insight into the nature of motivational processes at work, and on the other hand, it uncovers what specific behaviors leaders should exhibit to increase work engagement. That is the reason why a research program into engaging leadership was started a few years ago, the first results of which are discussed in this article.

It should be noted in advance that it is definitely *not* the intention to introduce yet another leadership concept, because there already are too many of them. Instead of starting with leadership behavior and then examining its effects on employee motivation and performance, we turned it around—so to speak—and started with work engagement and asked ourselves; what kind of leadership behaviors can promote work engagement among employees? In doing so, we follow the recommendations of Bormann and Rowold (2018) to further prevent the proliferation of leadership concepts. They argue for narrow instead of broad approaches that focus on specific employee behaviors—such as work engagement—rather than on a very broader range of outcomes. We also take a second recommendation to heart, namely that leadership concepts should have a solid theoretical foundation, for which Self-Determination Theory (SDT) seems to be particularly suitable.

**Figure 1:**
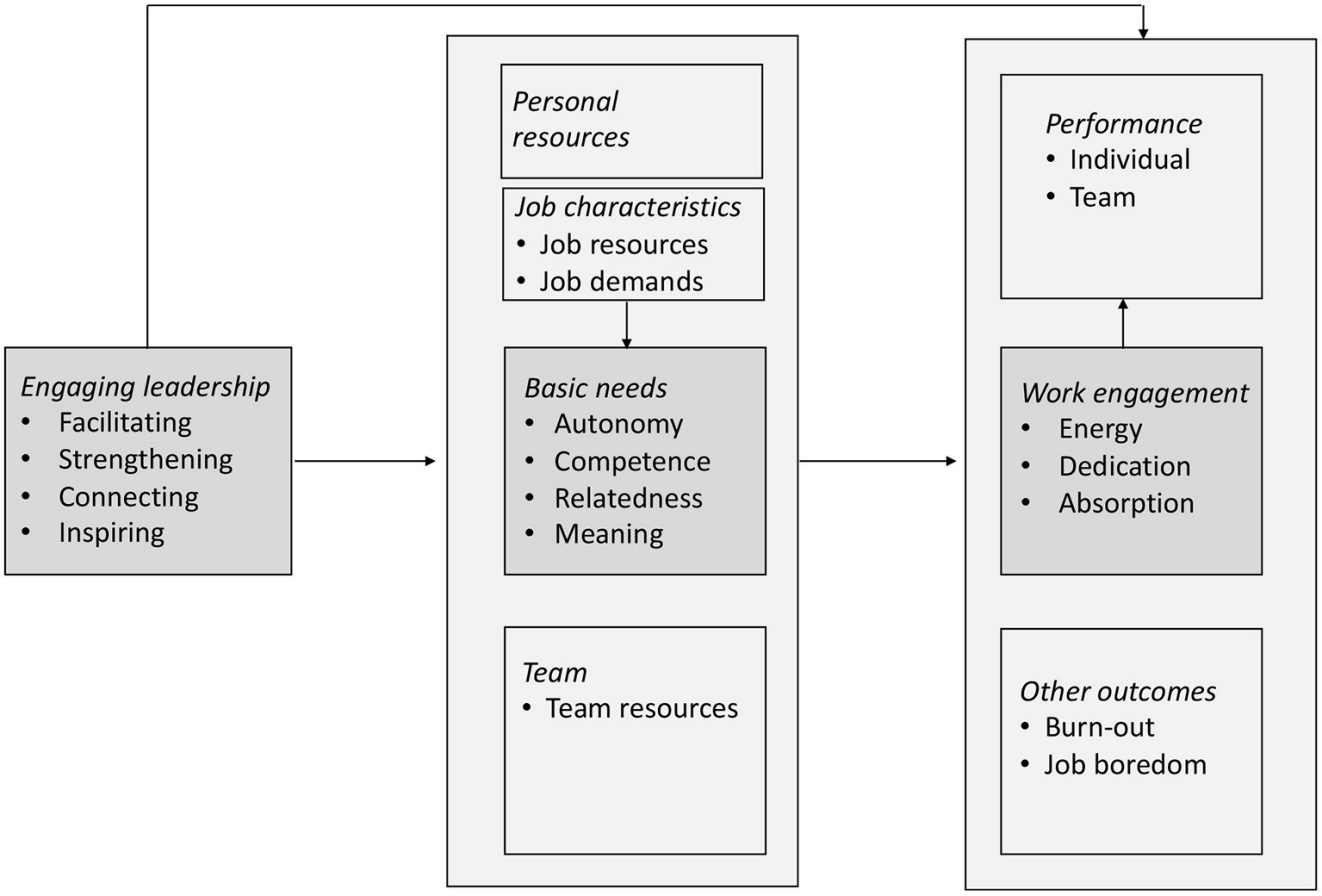
Engaging leadership in the work context.

This paper consists of three sections. In the first section, the concepts of work engagement and engaging leadership are explained, including their relationships with self-determination. Also, the difference with transformational leadership is discussed as well as the way in which engaging leadership is assessed. In the second section, the empirical results of recent studies on engaging leadership are reviewed, which are graphically summarized in Figure 1. Finally, the paper is closed with a concluding section, in which also the future of engaging leadership research is discussed.

## The Concepts of Work Engagement and Engaging Leadership

### Work Engagement

What exactly is work engagement? The most common description reads: “a positive, fulfilling, work related state of mind that is characterized by vigor, dedication, and absorption, whereby vigor refers to high levels of energy and mental resilience while working, the willingness to invest effort in one's work, and persistence even in the face of difficulties; dedication refers to being strongly involved in one's work, and experiencing a sense of significance, enthusiasm, inspiration, pride, and challenge; and absorption refers to being fully concentrated and happily engrossed in one's work, whereby time passes quickly and one has difficulties with detaching oneself from work” (Schaufeli et al., 2002, p. 74). Based on this definition, the Utrecht Work Engagement Scale (UWES; Schaufeli et al., 2006), has been developed, which is used in almost 90 percent of all scientific papers on work engagement (Bailey et al., 2017).

Just to clear up a misconception right away; work engagement differs from work addiction, which it is often confused with (Van Beek et al., 2011). Although similar to workaholics engaged employees work hard as well, their motivation differs fundamentally. Engaged employees invest highly in their job because they enjoy it, nevertheless they know when to stop and also have a private life outside of their work. In contrast, workaholics work so hard because they have no other choice; they are driven by an irresistible inner need to work, and when they don't, they feel useless, nervous, uneasy, restless and guilty. Therefore, it has been argued that engaged employees have a positive (approach) motivation and workaholics a negative (avoidance) motivation (Taris et al., 2014). The former are attracted by work because it is fun, whereas the latter are driven to work in an attempt to avoid the negative thoughts and feelings that are associated with *not* working.

Two decades of research has unequivocally demonstrated that engagement is good for employees as well as for the organizations they work for (for reviews see: Schaufeli, 2014; Schaufeli and Bakker, 2020). For example, engaged employees suffer less from depression and all kinds of other stress complaints, and run a lower risk of cardiovascular disease. Hence, their sickness absenteeism is lower than that of their less engaged co-workers. Engaged employees also feel strongly committed to their organization and therefore do not have the intention to leave. Furthermore, they like to learn and develop themselves, take personal initiative, and are innovative and make few mistakes. No wonder they perform better, also according their co-workers and supervisors. In addition, work engagement is also positively related to business results. For instance, engaged entrepreneurs achieve more growth and business success and engaged supervisors achieve better results with their teams. Engaged employees not only ensure higher financial turnover and productivity, but also provide better service. For example, a recent study of more than 100 publicly traded US companies showed that employee engagement predicts profitability and customer satisfaction over a period of 1–2 years (Schneider et al., 2018). In short, it is important to organizations for several reasons to promote employee engagement.

### Work Engagement and Self-Determination

Self-Determination Theory (SDT; Ryan and Deci, 2017; Ryan et al., 2021) is a general motivation theory, which has been studied in the context of school, education and sports as well as at work. Unlike most other motivational theories, the emphasis is not so much on the strength of motivation, but on its *quality*. According to SDT, high-quality motivation arises when three basic psychological needs are satisfied. First, the need for *autonomy*, which refers to the desire to be able to act psychologically freely. Employees feel autonomous when they can decide for themselves and make their own choices. Second, the need for *competence*, which refers to the desire to deal effectively with the environment. Employees feel competent when they can learn and develop, allowing them to adapt flexibly to what the work demands of them. Third, the need for *relatedness*, which refers to the desire to build positive relationships with others, to feel loved and cared for, and to care for others oneself. Employees feel connected when they are part of a close-knit team that supports each other and shares personal feelings and thoughts.

According to SDT, these three basic needs are innate and are, as it were, ingrained in human nature. It is therefore not a question of whether these needs are present or not—after all, everyone possesses them—but to what *extent* they are *satisfied*. SDT posits that the satisfaction of these basic needs is just as essential for optimal psychological functioning of people as food is necessary for their physical health. When these three basic needs are met, high-quality motivation—in SDT dubbed autonomous motivational regulation—exists that ensures optimal functioning of employees, both in terms of well-being and performance. To what extent basic needs are actually satisfied ultimately depends on the work context, which “nurtures” these needs, as it were.

Congruent with SDT, it was found that the satisfaction of the three basic needs mediates the relationship between job resources and work engagement (Van den Broeck et al., 2008). When all three basic needs were met so that employees are able to decide about the tasks they have to perform (autonomy), can use their skills (competence), and receive positive feedback from others at work (relatedness), they feel engaged. Another study showed a positive relationship between need satisfaction and work engagement as well, this time followed by better work performance in the form of extra-role behavior (Van Beek et al., 2014). That research also showed that work addiction may develop when basic needs are *no*t met. Finally, it appears that engagement is positively related to the extent to which basic needs are satisfied, also when controlling for the big-five personality traits (Sulea et al., 2015). If these needs are *not* met, students feel bored and also report burnout complaints, this research shows.

In summary, as predicted by SDT, it appears that the satisfaction of basic psychological needs at work is associated with optimal functioning, in terms of work engagement and job performance. When those needs are *not* met, suboptimal functioning might occur, such as work addiction, boredom or burnout. Finally, it appears that rather than on people's personality, the satisfaction of their basic needs depends on the characteristics of their jobs.

### Engaging Leadership and Self-Determination

Now that we know that employees are more engaged the more their basic needs are met, the question is how leaders can contribute to this. In other words, what kind of leadership behaviors should they exhibit in order to fulfill their followers' basic needs? Schaufeli (2015a) argued that “engaging leaders” may satisfy the need for autonomy, competence and relatedness of their followers by facilitating (empowering), strengthening and connecting them, respectively. By *facilitating* employees, for example by granting freedom and responsibility and giving them voice, they feel psychologically free to make their own decisions. This boils down to empowering employees. As a result, facilitating leaders satisfy the need for autonomy of their followers. By *strengthening* employees, for example by delegating tasks and responsibilities, giving them challenging jobs and stimulating their talents, they will feel more competent. Strengthening refers particularly to acquiring or increasing knowledge and skills. Leaders who strengthen their followers therefore satisfy their need for competence. Finally, by *connecting* team-members, for example by encouraging collaboration and creating a good team spirit, they will feel more comfortable and experience a sense of togetherness. Hence, leaders who connect their followers satisfy their basic need for relatedness.

A fourth basic need has been added on the basis of both theoretical and practical considerations; the need for meaning. This refers to the desire to perform useful and important work that contributes to something beyond one's own person, thereby transcending oneself. Employees experience that their work is meaningful when they feel that their contribution makes a difference. Frankl (1946) and Baumeister (1991) have convincingly argued that the need for meaning can be considered a basic human need as well. It seems that particularly in the public sector (e.g., among teachers and health care workers), meaningful work is important for work engagement (Mostafa and El-Motalib, 2018). In SDT, the basic need for meaning is implicitly understood as part of the need for autonomy. After all, true psychological freedom (autonomy) only exists when choices and decisions are based upon one's own personal values. If I can only choose from alternatives that are not meaningful to me—i.e., don't align with my own personal values—I will feel compelled to do something I don't really want to. From interviews with employees, we learned that performing meaningful work was very important to them and that leaders could stimulate meaningfulness by inspiring them. They can do this, for example, by enthusing their followers for a certain vision, mission, idea or plan and by acknowledging their personal contribution to the overall goal of the team or the organization. In short, *inspiring* leaders satisfy their employees' need for meaningfulness.

In summary, engaging leadership is defined as leadership behavior that facilitates, strengthens, connects and inspires employees in order to increase their work engagement. By facilitating, strengthening, connecting and inspiring, the employees' basic psychological needs for autonomy, growth, connectedness and meaning are satisfied, respectively, which in their turn, increases their work engagement.

### Engaging and Transformational Leadership

As such, engaging leadership bears a certain resemblance to transformational leadership (Bass, 1985), which is currently the most frequently studied leadership concept. Transformational leadership also consists of four aspects; (1) inspiration, i.e., motivation through charisma; (2) intellectual stimulation, i.e., encouraging innovation and creativity; (3) idealized personal influence, i.e., acting as a role model; (4) individual consideration, i.e., coaching, support and advice. The first two leadership behaviors more or less correspond to inspiring and facilitating, respectively, of engaging leadership. However, acting as a role model and individual consideration are *not* part of engaging leadership. Rather than merely exhibiting exemplary behaviors as a role model, engaging leadership is about actively stimulating employees' sense of autonomy, competence, relatedness and meaning. Furthermore, engaging leadership is about team consideration (i.e., stimulating connectedness and togetherness of team members), instead of individual consideration (i.e., personal support assistance and advice), as is the case with transformational leadership. Conversely, connecting and strengthening (promoting employee's strengths and talents), are not included in transformational leadership. A recent meta-analysis found a moderate positive correlation (*r* = 0.42) between transformational leadership and work engagement across 86 studies (DeCuypere and Schaufeli, 2021).

Van Knippenberg and Sitkin (2013) have severely the criticized the transformational leadership concept up to the point that that they advised “going back to the drawing board.” Their main criticism focuses on the inadequate definition of the four elements of transformational leadership and the lacking theoretical foundation of the concept. Hence, we heeded their call to go back to the drawing board and start from theory. This resulted in the concept of engaging leadership that is firmly rooted in Self-Determination Theory. However, this does not alter the fact that a certain overlap exists between both leadership concepts, so that it is not surprising that a consistent, positive relationship is found between transformational leadership and work engagement (Carasco-Saul et al., 2015). On the other hand, however, transformational and engaging leadership can, in fact, be distinguished empirically. For example, a study among Australian employees found that engaging and transformational leadership each loaded on a separate factor instead of collapsing into one common factor (Smith, 2018). Furthermore, another study showed that both engaging and transformational leadership explained independently from each other similar amounts of variance of work engagement of Indonesian employees (Rahmadani and Schaufeli, 2020).

In summary; transformational and engaging leadership partially overlap, which explains that both independently correlate positively with work engagement. Theoretically speaking, however, engaging leadership is superior because it is firmly rooted in a widely recognized theory of motivation that describes the psychological mechanism through which leadership leads to work engagement. In section Empirical Research on Engaging Leadership we will discuss the empirical support for this claim.

### The Measurement of Engaging Leadership

Employees' perception of engaging leadership can be measured using a short self-report questionnaire consisting of three items for each of the four aspects; the Engaging Leadership Scale (ELS; see Appendix). Several studies using confirmatory factor analysis confirmed that the ELS contains four components: inspiring, facilitating, strengthening, and connecting (Rahmadani and Schaufeli, 2020; Rahmadani et al., 2020a; Nikolova et al., 2021). However, these components are so closely related that, practically speaking, it is more convenient to use the total score of the ELS. In addition, a 360-degree study that included focal leader's followers, superiors and fellow-team leaders provided strong evidence of a halo effect (Robijn, 2021a). This means that when a leader is positively assessed on one particular aspect of engaging leadership by a follower, superior or fellow-team leader, it is highly likely that this also applies to the remaining three aspects. This finding too points in the direction of using the total ELS-score. The 360-degree study also showed that the interrater agreement between followers and superiors of the focal leaders was much stronger (*r* = 0.42) than that between their followers and fellow-team leaders (*r* = 0.22). Perhaps this is due to the fact that both followers and superiors have a formal relationship with the focal leader, with the former receiving daily guidance and supervision and the latter directing and appraising team leaders.

It is important for the validity of the ELS that team members agree in their assessment of the level of engaging leadership of their team leader. Indeed, this appears to be the case, which means that team members share their perception of engaging leadership so that the individual scores of team members can be aggregated at the team level (Rahmadani et al., 2020b; Salas-Vallina et al., 2021; Mazetti and Schaufeli, under review). Based on such aggregated team scores teams can be differentiated according to the level of engaging leadership as perceived by their members. Accordingly, this type of data-aggregation makes it possible to examine engaging leadership not only as an individual perception but also as a team characteristic (see section Team-Level Engaging Leadership).

Finally, the ELS is a reliable measurement tool with both a high internal consistency (α > 0.85; Rahmadani et al., 2019; Rahmadani and Schaufeli, 2020; Salas-Vallina et al., 2021) and a stability of 0.79 and 0.52 after 6 months (Smith, 2018) and 1 year (Nikolova et al., 2019), respectively. In summary; the ELS has good psychometric properties and can therefore be used as valid and reliable indicator of perceived engaging leadership, both at individual as well as team level.

## Empirical Research on Engaging Leadership

Below the results of recent investigations about engaging leadership are reviewed. First studies on the mediation of basic need fulfillment will be discussed, followed by studies on the mediation of job characteristics and personal resources. Special attention is paid to engaging leadership at team level. This section is closed with a figure that summarizes the main results of studies.

### Mediation of Basic Psychological Needs

As argued above and based on SDT, engaging leadership is expected to lead to the satisfaction of basic psychological needs and subsequently to an increase in employee's work engagement. In other words, satisfaction of the four basic needs should mediate the effect of engaging leadership on work engagement. This indeed appears to be the case, for example among South African miners (Erasmus, 2018), employees of Flemish health insurance funds (Robijn et al., 2020), office staff of a Dutch insurance company (Robijn, 2021b), staff of the back office of a Dutch technology company (Van Tuin et al., 2020a) and finally Russian civil servants and employees working at an Indonesian palm oil plantation (Rahmadani et al., 2019). In all cases full mediation was observed, except for Russian civil servants and Dutch back-office staff, where the mediation was partial in nature. This means that in addition to an indirect relationship—*via* the satisfaction of basic needs—a significant *direct* relationship was also found between leadership and engagement. Furthermore, engaging leadership is also indirectly related—*via* the satisfaction of basic needs—to boredom (Erasmus, 2018) and team performance (Robijn, 2021b); when employee's basic needs are met, they feel less bored and perform better as a team.

A recent longitudinal study among Indonesian workers shows a more complex relationship between engaging leadership and engagement (Rahmadani et al., 2020a). This study observed that engaging leadership leads to the perception of more job resources 1 year later (e.g., a better person-job fit, more use of skills and feedback), which in turn, leads to the fulfillment of basic needs and subsequently to more work engagement. Thus, engaging leaders satisfy their increase employees' basic psychological needs not only directly, but also indirectly, though increasing their job resources. Another longitudinal study also found that engaging leadership leads to an increase in job resources, such as autonomy and social support, over a period of 1 year among Dutch hotel employees (Nikolova et al., 2019). However, this study did not include basic psychological needs and energy resources did not appear to mediate the relationship between engaging leadership and job resources.

### Mediation of Job Characteristics and Personal Resources

In some other studies, however, a mediation effect of job resources (and job demands) was found. For example, Schaufeli (2015a) showed in a representative sample of the Dutch working population that engaging leadership is positively associated with work engagement through job resources (e.g., team spirit, task variation and role clarity). At the same time, burnout is negatively associated with engaging leadership via lower work demands (e.g., work overload, emotional demands and work-home interference). Both mediation effects were replicated in a longitudinal study among employees of a Dutch government agency (Schaufeli, 2017). It was observed that engaging leadership led to more job resources and less job demands a year later, which in turn, was related to more work engagement and less burnout, respectively.

In addition to job characteristics (job demands and job resources) engaging leadership also has a positive impact on personal resources, such as optimism, resiliency, flexibility and self-efficacy, as was shown by another study that is based on the aforementioned representative Dutch sample (Schaufeli, 2015b). In their turn, these personal resources were positively associated with engagement. In other words, just like job resources, personal resources also appear to mediate the relationship between engaging leadership and work engagement (and burnout). This was confirmed in a longitudinal study of Dutch civil servants, that found that engaging leadership led to an increase in personal resources (i.e., optimism, resiliency, flexibility, and self-efficacy) over a period of 1 year, which in turn, led to more work engagement (Mazetti and Schaufeli, under review).

### Team-Level Engaging Leadership

The longitudinal study by Mazetti and Schaufeli (under review) not only looked at the effect of perceived engaging leadership on individual work engagement, but also at the effect at team level. It appeared that a year after baseline measurement, teams led by engaging leaders perceived more team resources (e.g., better communication and more participation in decision-making) compared to teams that that were led by less engaging leaders. In teams with more job resources, team members felt—in turn—more engaged than in teams with fewer job resources. In other words, it seems that also at team level job resources mediate the relationship between engaging leadership and work engagement. Engaging leadership is therefore not only important for individual employees, but also for teams. This is confirmed by another longitudinal Indonesian study, which showed that employees from teams led by engaging leaders not only feel more engaged individually, but also collectively as a team experience more work engagement (Rahmadani et al., 2020b). This, in turn, led individual team members to performing better, learn more and display more innovative work behavior. But also, teams learned more collectively and showed more innovative behavior as a team. In other words, engaging leaders boost individual and team performance by increasing employee engagement.

Finally, a recent Spanish study investigated to what extent engaging team leadership enhances over time the effect of HR policies regarding job security, training and education, quality of work, teamwork, communication and leadership (Salas-Vallina et al., 2021). As expected, it was observed 1 year later that the effects of these policies were more positive in teams led by engaging team leaders, as compared to teams with a less engaging leaders. For example, team members from teams led by an engaging leader felt happier and less exhausted and had more trust in leadership. In turn, happiness and trust contributed positively to team member's performance and negatively to feelings of exhaustion. In other words, HR policies seem to have positive effects on the well-being and performance of team members, particularly when the team is led by an engaging leader.

### Summary of Results

Figure 1 summarizes the results of engaging leadership research; the core assumption of SDT about the mediation of basic need satisfaction is shaded in gray. Although the arrows suggest causality, this has not (yet) been empirically confirmed in all cases; the issue of causality is further discussed in section Causality and Dynamic Relationships.

Generally speaking, there seem to be two avenues through which engaging leadership may promote work engagement: a direct and an indirect avenue, through satisfying follower's basic needs and increasing their job, team and personal resources. Engaging leadership not only impacts work engagement, but is also negatively associated with burnout and boredom and positively with individual and team performance. There are also indications for more complex relationships in which engaging leadership, through increasing job resources, contributes the fulfillment of basic needs and subsequently to work engagement. Furthermore, engagement appears to be positively related to individual and team performance, as was already known from previous research (see section Work Engagement).

## Conclusions and Outlook

This article attempts to provide an overview of recent research about engaging leadership, a novel leadership concept that is developed using SDT, specifically with the aim to uncover how leaders may promote work engagement among their followers. As hypothesized, engaging leaders promote work engagement by satisfying their employees' basic psychological needs for autonomy, competence, relatedness and meaning. They do so by facilitating, strengthening, connecting and inspiring them. In addition to satisfying basic needs, engaged leaders also reduce job demands and increase job and team resources as well as personal resources.

That means that when employees feel autonomous, competent and connected to their co-workers and when they experience their work to be meaningful, they are less likely to suffer from job stress and can tap better into job resources. This energizes them and boosts their work engagement, which in its turn, has a beneficial impact on their job performance, including learning ability and innovativeness. In addition, it appears that the more the basic needs are satisfied, the fewer burnout and boredom at work is experienced. Importantly, engaging leaders affect not only individual employees but the entire team, as they help the team to function better by increasing team resources, thereby fueling a collective sense of team engagement that in turn leads to better team performance. Engaging team leaders also enhance the positive effect of the organization's HR policies on the well-being and performance of employees. In that sense, engaging leaders are a crucial link between top management and the shopfloor.

It can therefore be concluded that, so far, research results regarding engaging leadership are encouraging. Moreover, many findings—such as the pivotal role of basic need satisfaction—have been replicated in different occupational groups from various countries. This means that many results can be generalized across work situations, occupations and countries. The questionnaire that is designed to measure engaging leadership (ELS) also appears to be reliable and valid, both at individual and team level. Despite these encouraging findings, five themes remain unexplained, which together form an agenda for future research on engaging leadership.

### Causality and Dynamic Relationships

Much of the research which is discussed above is cross-sectional in nature, so that no conclusions can be drawn about the direction of the relationships. That the observed relationships are not always in the expected direction illustrates a longitudinal study by Nikolova et al. (2019). This study found that the employees' current level of work engagement predicts their leader's future level of engaging leadership, rather than the other way around. This could indicate that there is a dynamic process in which engaging leadership and work engagement mutually influence each other. A dynamic, bi-directional process like this should be investigated further using longitudinal research with at least three measurement occasions. Accordingly, more longitudinal research on engaging leadership is needed, especially when it comes to mediation.

Another method to unravel dynamic, bi-directional relationships is diary research, in which employees fill out a short questionnaire every day for a period of 1 or 2 weeks. This is based on the assumption that the level of engaging leadership varies from day to day. A Finnish diary study among care workers showed that especially on days when leaders tried to connect their team members, more job crafting was observed (Mäkikangas et al., 2017). This means that particularly on those days care workers tried to bring their work in line with their own preferences. This diary study illustrates that team leaders may stimulate proactive behavior such as job crafting by connecting team members; i.e., by satisfying their need for relatedness.

### Disengaging Leadership

Engaging leadership can be contrasted with its opposite disengaging leadership, which is characterized by: (1) coercing (i.e., authoritarian behavior that restricts and controls employees); (2) eroding (i.e., obstructing employee's professional development and diminishing their sense of competence) (3) isolating (i.e., disconnecting employees from the rest of the team and pitting them against each other); (4) demotivating (i.e., creating, among employees, an image that their job is meaningless and their work does not contribute to anything important). This way, the basic needs for autonomy, competence, relatedness, and meaning are thwarted. Frustration of basic needs fundamentally differs from not satisfying them because it involves active thwarting rather than not stimulating and letting it run its course (Vansteenkiste and Ryan, 2013). A first study on disengaging leadership indeed showed—as expected—that it negatively relates to engaging leadership and satisfaction of basic needs, and positively to need frustration (Nikolova et al., 2021). In addition, this study found that the positive relationship between disengaging leadership and emotional exhaustion (the core component of burnout) was mediated by a frustrated need for autonomy. In other words, disengaging leaders thwart the need for autonomy, leaving employees feeling exhausted. In fact, this negative process is analogous to what is depicted in Figure 1 for positive, engaging leadership behavior. More research is needed to uncover the extent to which other variables from Figure 1 play a similar—but opposite—role in case of disengaging leadership.

### The Measurement of Engaging Leadership

The psychometric features of the ELS are encouraging as evidenced by its internal consistency, stability and factorial validity. There is also a reasonable correlation between self-assessment and the assessment of others, and it appears that engaging leadership, as measured by the ELS, can be distinguished from transformational and disengaging leadership. The concept validity of the ELS is supported by the research findings that are summarized in Figure 1. Finally, some indications for discriminant validity vis-à-vis transformational leadership were found. However, what is still lacking is validation based on actual leadership behavior. After all, research to date was about employee's perception of leadership, based on the adage that engaging leadership is in the eye of the beholder. It appears from 360-degree assessments that followers, superiors and fellow-team leaders reasonably agree about the extent to which the focal leader displays engaging leadership behavior (Robijn, 2021a). This in itself is encouraging, albeit that the self-perception of leaders corresponds much less closely with the assessment of others around him or her. By using behavioral observations, for instance behaviorally anchored rating scales, a better picture may be obtained of specific engaging leadership behaviors. How exactly does facilitating, strengthening, connecting and inspiring look like in practice?

### Interventions

Because engaging leadership is beneficial for organizations, it makes sense to integrate it into management development programs. But what do we know about the effectiveness of interventions to improve engaging leadership? A first study into the effect of an 8-month engaging leadership training, which consisted of six monthly training days, supplemented by two face-to-face coaching sessions and three peer consultation sessions, yielded mixed results (Van Tuin et al., 2020b). The training days included: introduction and discussion about the goal of the program (day 1); explanation about engaging leadership and setting personal goals (day 2); discussing the administered team questionnaires and monitoring the progress regarding one's personal goals (day 3); increasing resilience and dealing with negative emotions (day 4); motivational coaching based on the SDT (day 5); evaluation of the program including the achievement of one's personal goals (day 6). Results of the study showed on the one hand that the level of engagement in teams of leaders who had participated in the intervention program had *not* increased significantly compared to control teams whose team leaders had not participated in the intervention. On the other hand, however, absenteeism decreased and productivity of the intervention teams increased compared to the control teams. Quite importantly, both positive effects were still observed 6 months after the intervention had finished. Perhaps it may take a relatively long time before a positive effect on work engagement of team members can be observed. Unfortunately, in this study, work engagement was only measured before and immediately after the intervention and not after another 6 months. Most likely, during the intervention period, engaging leaders increased their team's resources, thereby reducing absenteeism rates and increasing team productivity. Either way, more research is needed to unravel how the positive effects of leadership interventions come about.

### All Roads Lead to Rome

It is also important to investigate alternative psychological processes that may explain why engaging leaders promote work engagement. In a theoretical contribution, DeCuypere and Schaufeli (2020) described five pathways through which positive leadership behavior can influence engagement; it seems that all five roads lead to Rome. Only the two indirect paths have been explored to date; the “motivational” (satisfaction of basic psychological needs) and the “material” path (improvement of work characteristics). In addition, the authors distinguish three other, *direct* paths, namely an affective, cognitive and behavioral path. These could potentially explain the direct relationship between engaging leadership and work engagement as shown in Figure 1. The *affective* path involves emotional contagion, meaning that positive emotions of leaders automatically cross-over to their followers in the form of work engagement. The direct *cognitive* path involves social exchange, meaning that employees feel obligated, as it were, to reciprocate the commitment of their engaging leader by engaging similarly in their work. Finally, in the direct *behavioral* path social learning plays a key-role, meaning that the engaging leader acts as a role model. Future research on these three direct, alternative pathways may elucidate other psychological mechanisms underlying engaging leadership.

## Final Note

Recent research on engaging leadership did yield valuable insights about the positive effects for employees and teams. The concept of engaging leadership is not only scientifically interesting because it unveils the motivational dynamic at the workplace, but also important for organizational practice because engaging leadership produces positive outcomes.

Moreover, a practical advantage that helps implementing engaging leadership in organizations is its intuitive appeal. It hardly needs any explanation for executives, managers and HR-officers that leaders who facilitate, strengthen, connect and inspire allow their employees to flourish and grow and therefore achieve superior results. The fact that this seems to be confirmed by scientific research is an additional bonus. After all, not everything that has intuitive appeal in organizations can withstand scientific scrutiny.

## Author Contributions

The author confirms being the sole contributor of this work and has approved it for publication.

## Funding

This study was supported by Onderzoeksraad KU Leuven: BOFZAP-14/001.

## Conflict of Interest

The author declares that the research was conducted in the absence of any commercial or financial relationships that could be construed as a potential conflict of interest.

## Publisher's Note

All claims expressed in this article are solely those of the authors and do not necessarily represent those of their affiliated organizations, or those of the publisher, the editors and the reviewers. Any product that may be evaluated in this article, or claim that may be made by its manufacturer, is not guaranteed or endorsed by the publisher.

## Appendix

### The Engaging Leadership Scale (ELS)^©^

*Instruction*

The following 12 statements are about your supervisor. Please read each statement carefully and decide how you feel about him or her. Check the box that best describes your present agreement or disagreement with each statement below.

**Table:**

**Completely disagree**	**Disagree**	**Neither agree nor disagree**	**Agree**		**Completely agree**	
**1**	**2**	**3**	**4**		**5**	
**My supervisor…**		**1**	**2**	**3**	**4**	**5**
**Strengthening**						
1. …encourages team members to develop their talents as much as possible		□	□	□	□	□
2. …delegates tasks and responsibilities to team members		□	□	□	□	□
3. …encourages team members to use their own strengths		□	□	□	□	□
**Connecting**						
4. …encourages collaboration among team members		□	□	□	□	□
5. …actively encourages team members to aim for the same goals		□	□	□	□	□
6. …promotes team spirit		□	□	□	□	□
**Empowering**						
7. …gives team members enough freedom and responsibility to complete their tasks		□	□	□	□	□
8. …encourages team members to give their own opinion		□	□	□	□	□
9. …recognizes ownership of team member's contributions		□	□	□	□	□
**Inspiring**						
10. …is able to enthuse team members with his/her plans		□	□	□	□	□
11. …makes team members feel that they contribute to something important		□	□	□	□	□
12. …is inspiring		□	□	□	□	□

*© Schaufeli (2015b). The Engaging Leadership Scale (ELS) is free for use for non-commercial, scientific research. Commercial and/or non-scientific use is prohibited, unless previous written permission is granted by the author*.
